# Case–control study of lifetime alcohol consumption and endometrial cancer risk

**DOI:** 10.1007/s10552-013-0275-0

**Published:** 2013-08-09

**Authors:** Christine M. Friedenreich, Thomas P. Speidel, Heather K. Neilson, Annie R. Langley, Kerry S. Courneya, Anthony M. Magliocco, Linda S. Cook

**Affiliations:** 1 Department of Population Health Research, Cancer Control Alberta, Alberta Health Services, Box ACB, 2210-2nd St SW, Calgary, AB T2S 3C3 Canada; 2 Department of Community Health Sciences, Faculty of Medicine, University of Calgary, 3330 Hospital Drive NW, Calgary, AB T2N 4N2 Canada; 3 Department of Oncology, Faculty of Medicine, University of Calgary, 1331 29 St NW, Calgary, AB T2N 4N2 Canada; 4 Faculty of Physical Education and Recreation, University of Alberta, E488 Van Vliet Centre, Edmonton, AB T6G 2H9 Canada; 5 Department of Anatomical Pathology, H. Lee Moffitt Cancer Center, 12902 Magnolia Drive, Tampa, FL 33612 USA; 6 Department of Internal Medicine, NM Health Sciences Center, University of New Mexico, MSC 10 5550, 1 University of New Mexico, Albuquerque, NM 87131-0001 USA

**Keywords:** Endometrial cancer, Alcohol drinking, Ethanol, Beer, Wine, Liquor

## Abstract

**Purpose:**

Alcohol consumption is hypothesized to increase the risk of endometrial cancer by increasing circulating estrogen levels. This study sought to investigate the association between lifetime alcohol consumption and endometrial cancer risk.

**Methods:**

We recruited 514 incident endometrial cancer cases and 962 frequency age-matched controls in this population-based case–control study in Alberta, Canada, from 2002 to 2006. Participants completed in-person interviews querying lifetime alcohol consumption and other relevant health and lifestyle factors. Participants reported the usual number of drinks of beer, wine, and liquor consumed; this information was compiled for each drinking pattern reported over the lifetime to estimate average lifetime exposure to alcohol.

**Results:**

Lifetime average alcohol consumption was relatively low (median intake: 3.9 g/day for cases, 4.9 g/day for controls). Compared with lifetime abstainers, women consuming >2.68 and ≤8.04 g/day alcohol and >8.04 g/day alcohol on average over the lifetime showed 38 and 35 % lower risks of endometrial cancer, respectively (*p* trend = 0.023). In addition, average lifetime consumption of all types of alcohol was associated with decreased risks. There was no evidence for effect modification by body mass index, physical activity, menopausal status, and hormone replacement therapy use combined and effects did not differ by type of endometrial cancer (type I or II).

**Conclusion:**

This study provides epidemiologic evidence for an inverse association between relatively modest lifetime average alcohol consumption (approximately 1/4 to 1/2 drink/day) and endometrial cancer risk. The direction of this relation is consistent with previous studies that examined similar levels of alcohol intake.

## Introduction

Endometrial cancer is hypothesized to develop in response to high levels of estrogens unopposed by adequate levels of progesterone [1]. In epidemiologic studies, alcohol consumption has been associated with increased levels of circulating estrogens in pre- and post-menopausal women, and may therefore increase the risk of endometrial cancer [2–6]. Numerous epidemiological studies have investigated the relation between overall alcohol consumption and endometrial cancer risk; results are varied showing, for some levels of consumption, statistically significant positive [7, 8] and inverse associations [9–16] or in most cases no relation [17–36]. Only a few of those studies, however, attempted to capture lifetime alcohol intake in their exposure assessments [25, 26, 29, 36]. Given inconclusive findings and the paucity of information regarding the impact of alcohol intake over the lifetime on endometrial cancer risk, we conducted this population-based case–control study to assess specifically how alcohol consumption over the entire lifetime influences endometrial cancer risk and to investigate the role of different types and doses of alcohol on risk.

## Materials and methods

### Study population

A population-based case–control study was conducted in Alberta, Canada, from 2002 to 2006 to examine the association between physical activity and endometrial cancer [37]. The Alberta Cancer Registry and pathology reports were used to recruit incident, histologically confirmed, primary endometrial cancer cases (*n* = 549). Random digit dialing [38] was used to accrue population-based controls (*n* = 1,036) who were frequency-matched to cases by age (±5 years) in a 2:1 ratio. Response rates of 67.9 % for cases and 52.2 % for controls were achieved. For the cases, of the 808 who were eligible and received physician approval, 249 women declined participation, seven could not be contacted, and three were not endometrial cancer cases upon re-review resulting in 549 eligible for this analysis. For the controls, of the 1,988 eligible women identified, 14 were excluded for no further contact and 948 refused participation leaving 1,036 who completed the interview. Participants had no previous cancer history (excluding non-melanoma skin cancer), and controls had no history of hysterectomy or endometrial ablation. Women were excluded if interviews were unsatisfactory (*n* = 7 cases, *n* = 4 controls), or if they were missing covariate data used in multivariable modeling (*n* = 28 cases, *n* = 70 controls). One participant’s beer consumption was set to missing for one time period because of unreasonably high reported consumption. The final analytic sample comprised 514 cases and 962 controls. All participants provided informed written consent, and ethical approval for the study was obtained from the Alberta Cancer Board and the University of Calgary Ethics Review Boards.

### Data collection

Information regarding demographic characteristics, endometrial cancer risk factors, lifetime total physical activity levels, smoking history, and hormone use was collected through structured in-person interviews. Recall of previous activity patterns and lifestyle behaviors was facilitated through the use of cognitive interviewing methods [39, 40]. Anthropometric measures (height, weight, waist, and hip circumferences) were taken in three repeat measurements in a standardized fashion following the interview with mean values of each measure used for analysis.

Regarding alcohol consumption, participants were asked whether or not they had consumed six or more drinks of beer, wine, or liquor in any given year of their life before an assigned reference date (date of diagnosis for cases and a comparable date for controls) in order to identify women who have rarely consumed alcohol throughout their lives. These women were classified as lifetime abstainers. Women who answered yes to this question were asked the usual number of drinks of beer (12 oz bottles or cans/360 ml), wine (5 oz/140 ml), and liquor (1.5 oz/45 ml) consumed per week or per month. This information was collected for each pattern of drinking over each woman’s lifetime with ages at the beginning and the ending of each pattern recorded. Participants who reported a pattern that started and ended in the same year were assigned 1 year of consumption.

### Statistical analysis

Unconditional logistic regression analyses were conducted to estimate odds ratios (OR) and 95 % confidence intervals (CI) for the risk of endometrial cancer in relation to the levels of mean daily grams of alcohol intake from all alcoholic beverages combined and for each alcohol type (beer, wine, and liquor) individually. We converted the volumes of reported alcohol intake into grams of alcohol based on the conversion of 13.6 g of alcohol per standard drink that was recorded [41]. Continuous alcohol consumption in 1 g per day and 6.8 g per day increments (equivalent to ½ a standard drink in Canada) for all types of alcohol combined and each type individually was analyzed. In addition, mean daily grams of alcohol intake averaged over the lifetime were analyzed categorically in tertiles based on the distribution of drinking among the controls. The likelihood ratio test was used to assess the overall contribution of a categorical variable to the model. To address the timing of alcohol exposure, women were categorized as a lifetime abstainer, former drinker (in our study, these women quit drinking 2–58 years before reference date; median 17 years), or current drinker as of the reference date. In addition, lifetime abstainers were compared to ‘ever’ drinkers, defined as women reporting six or more drinks in 1 year over a lifetime. Four additional models estimated OR and 95 % CI for different life periods of alcohol consumption (≤17, 18–34, 35–50, and ≥51 years of age); alcohol intake was analyzed both as a continuous variable and by tertiles within each age period. The continuous alcohol intake measures were modeled using an extra parameter to account for whether or not a participant was ever exposed, thus taking into account the common occurrence of unexposed participants in a continuous measurement [42]. For all models, potential covariates were identified a priori, and those that were deemed of special interest (based on subject matter knowledge) were assessed for both confounding and effect modification.

Covariates that were identified were as follows: age (years), parity (nulliparous vs. multiparous), hormone replacement therapy (HRT) and menopausal hormones (estrogen, estrogen + progesterone, other, none versus post-menopausal status, and no HRT), oral contraceptive use (ever vs. never), residential status (rural vs. urban), type II diabetes, hypertension (yes vs. no), comorbidities (none, 1, ≥2 among: thrombosis, pulmonary embolism, myocardial infarction, stroke, and history of high cholesterol), mean lifetime MET-hours/week/year of physical activity, weight (kg), height (cm), hip circumference (cm), waist circumference (cm), glycemic load, fasting plasma glucose (mg/dl), cholesterol (mg), total dietary folate equivalent (mcg), insulin (mIU/l), and smoking status (current, ex-smoker, occasional vs. nonsmoker).

The bootstrap method was used to select a reduced model [43]. Covariates from the final models were assessed for confounding with all the other covariates in the model using the change in estimate method [44]. Our analyses by type of alcohol (beer/wine/liquor) included models that were adjusted for other types of alcohol intake. To assess linearity, we fitted restricted cubic splines [45, 46] for variables that were suspected to behave nonlinearly either based on previous evidence or on LOWESS plots of outcome versus each variable. As a result, age at reference date was modeled nonlinearly.

Effect modification was assessed for measured BMI (kg/m^2^) at reference date, mean total lifetime physical activity (MET-hours/week/year), and combined hormone therapy (HRT) use and menopausal status (a variable that combined levels for each type of HRT use (none, estrogen only, estrogen and progesterone, other) with peri- or post-menopausal status, vs. pre-menopausal and no HRT use) by fitting an interaction term between mean daily alcohol intake and each of these factors (one at a time) in the full model, followed by a likelihood ratio test. We also performed a polytomous logistic regression analysis to determine if the risks associated with alcohol intake differed by endometrial cancer type (I vs. II). Regression diagnostics and influence statistics were completed for final regression models to assess adherence to regression assumptions, and the Hosmer–Lemeshow goodness of fit test to assess model fit [47]. All statistical analyses were completed using Rstudio (Version 0.96.122, Boston, MA, USA) and Stata version 12 (StataCorp, College Station, TX, USA).

## Results

As previously reported [37], our study population had a mean age of 58 years, were primarily Caucasian (96 %), peri- or post-menopausal (88.9 %), and nonusers of HRT (58.1 %). On average, cases and controls were obese (median BMI = 31.0) and overweight (BMI = 27.2), respectively. Cases and controls were comparable on most characteristics, except that cases, on average, were heavier and had a greater median waist circumference. They were also more frequently nulliparous, type II diabetics, and had a higher self-reported prevalence of hypertension over their lifetime (Table 1).

**Table 1:** Distributions of selected characteristics among cases and controls, Alberta, Canada, 2002–2006 (*n* = 1,476)

Risk factor	Cases (*n* = 514)	Controls (*n* = 962)
	Median (25th, 75th percentile) or *n* (%)	Median (25th, 75th percentile) or *n* (%)
Age at reference date (year)	59 (53, 65)	59 (52, 66)
BMI (kg/m^2^)	31.0 (26.4, 36.8)	27.2 (24.1, 30.9)
Hip circumference (cm)	110.9 (102.6, 123.5)	104.8 (99, 112.5)
Waist circumference (cm)	95.5 (84, 108.6)	84.5 (76.5, 95.5)
Weight (kg)	81.1 (68.6, 98)	71.5 (63.1, 81.6)
Nulliparous	92 (17.9)	89 (9.3)
Menopausal status and hormone replacement therapy use combined (HRT)		
Peri- and post-menopausal, no HRT (referent category)	281 (54.7)	480 (49.9)
Peri- and post-menopausal, estrogen only	20 (3.9)	25 (2.6)
Peri- and post-menopausal, estrogen and progestin combined	132 (25.7)	322 (33.5)
Peri- and post-menopausal other hormones	27 (5.3)	26 (2.7)
Pre-menopausal	54 (10.5)	109 (11.3)
Smoking status		
Nonsmoker	263 (51.2)	487 (50.6)
Current smoker	68 (13.2)	116 (12.1)
Ex-smoker	162 (31.5)	339 (35.2)
Occasional smoker	21 (4.1)	20 (2.1)
MET-hours/week of lifetime total physical activity	100.4 (78.8, 126.5)	105.0 (82.8, 129.4)
Type II diabetes	62 (12.1)	51 (5.3)
Ever diagnosed with hypertension	161 (31.3)	171 (17.8)
Ever ≥6 alcoholic drinks per year over lifetime	407 (79.2)	819 (85.1)
Ever beer drinker	238 (46.3)	495 (51.5)
Ever wine drinker	337 (65.6)	695 (72.3)
Ever liquor drinker	358 (69.7)	699 (72.7)
Ethanol intake from any alcoholic beverage over lifetime (g/day)^a^	3.9 (1.2, 9.8)	4.9 (1.9, 11.3)
Ethanol intake from beer over lifetime (g/day)^a^	1.3 (0.4, 4.9)	2.2 (0.6, 4.9)
Ethanol intake from wine over lifetime (g/day)^a^	0.9 (0.3, 3.5)	1.7 (0.4, 4.5)
Ethanol intake from liquor over lifetime(g/day)^a^	1.3 (0.5, 3.9)	1.6 (0.5, 3.9)
Tertiles of ethanol intake over lifetime (g/day)^a,b^		
Lifetime abstainers	107 (20.8)	143 (14.9)
>0–≤2.68	165 (32.1)	273 (28.4)
>2.68–≤8.04	120 (23.4)	274 (28.5)
>8.04	122 (23.7)	272 (28.3)
Drink status		
Lifetime abstainer	107 (20.8)	143 (14.9)
Former drinker	90 (17.5)	137 (14.2)
Current drinker	317 (61.7)	682 (70.9)

^a^Lifetime ethanol intake was estimated as the mean of all self-reported alcohol consumption over an individual drinking lifetime

^b^For those who reported drinking alcohol, tertiles are based on the distribution of the controls

Lifetime average alcohol consumption was relatively modest in this study population with a median intake of 3.9 g/day (IQR 1.2, 9.8) and 4.9 g/day (IQR 1.9, 11.3) for cases and controls, respectively (Table 1). Analyzed as a continuous variable, alcohol consumption was not significantly associated with endometrial cancer risk among the drinkers in age- or multivariable-adjusted models (Table 2; multivariable-adjusted OR = 0.97, 95 % CI 0.89, 1.03 for every ½ drink increase in daily alcohol intake). However, when assessed by tertiles, (Table 2), the highest level of lifetime average alcohol consumption (>8.04 g/day) compared to lifetime abstainers was inversely associated with endometrial cancer risk (multivariable-adjusted OR = 0.65, 95 % CI 0.44–0.97, *p* trend = 0.023). Current and former drinking status compared to lifetime abstainers was also associated with decreased risks (multivariable-adjusted OR = 0.70, 95 % CI 0.51–0.98 and OR = 0.68, 95 % CI 0.45, 1.04, respectively). Ever being a drinker was also associated with a significant decreased risk (OR = 0.71, 95 % CI 0.52, 0.98). With the exception of former drinking, age-adjusted and multivariable-adjusted risk estimates were similar (Table 2). By the type of alcohol (Table 3), statistically significant inverse associations were found for the upper tertile of lifetime average wine consumption >3.29 g/day, above the middle tertile for average beer (>0.97 g/day) consumption, and for only the middle tertile of average liquor consumption (>0.80 and ≤2.94 g/day) compared to lifetime abstainers. Inverse trends were observed for all alcohol types (*p* trend ≤ 0.059). However, across all levels of consumption jointly, only lifetime beer consumption as a categorical variable was statistically significantly associated with decreased risk of endometrial cancer (*p* = 0.012 in the likelihood ratio test). While each model adjusted for intakes of other types of alcohol, the odds ratios and 95 % confidence intervals estimated without this adjustment were very similar (data not shown).

**Table 2:** Multivariable-adjusted odds ratios estimates (OR) and associated 95 % confidence intervals (CI) for lifetime average daily alcohol intake (*n* = 1,476)

Risk factor	Cases (*n*)	Controls (*n*)	Age-adjusted		Multivariable-adjusted	
			OR	95 % CI	OR^a^	95 % CI
Mean daily alcohol intake						
Per 1 g increase (continuous)^b^			1.00	0.99, 1.00	0.99	0.98, 1.00
Per 6.8 g increase (1/2 drink) (continuous)^b^			0.95	0.89, 1.01	0.97	0.89, 1.03
Ever drinker vs. lifetime abstainer	407	819	0.69	0.52, 0.92	0.71	0.52, 0.98
By tertiles^c,d^						
Lifetime abstainers	107	143	1.00	Referent	1.00	Referent
>0–≤2.68 g/day	165	273	0.81	0.59, 1.11	0.79	0.56, 1.13
>2.68–≤8.04 g/day	120	274	0.58	0.42, 0.82	0.62	0.42, 0.91
>8.04 g/day	122	272	0.60	0.43, 0.84	0.65	0.44, 0.97
*p* trend				0.001		0.023
Type of drinker^d^						
Lifetime abstainer	107	143	1.00	Referent	1.00	Referent
Former drinker^e^	90	137	0.88	0.61, 1.27	0.68	0.45, 1.04
Current drinker	317	682	0.62	0.47, 0.83	0.70	0.51, 0.98

^a^Adjusted for age at reference, nulliparous (vs. multiparous), HRT and menopausal hormones, rural residential status (vs. urban), hypertension, weight at reference, waist circumference, smoking status

^b^Among the alcohol drinkers

^c^For those who drink, tertiles are based on the distribution of the controls

^d^ *P* values were estimated for the likelihood ratio test of the overall importance of a categorical variable. For alcohol intake by tertiles *p* = 0.003, *p* = 0.046 for age- and multi-variable-adjusted models, respectively. For type of drinker, *p* = 0.002, *p* = 0.071 for age- and multi-variable-adjusted models, respectively

^e^A former drinker is defined as any participant who consumed more than six drinks in any given year up to 1 year before reference date

**Table 3:** Multivariable-adjusted odds ratios estimates (OR) and associated 95 % confidence intervals (CI) for lifetime average daily alcohol intake by type of drink (*n* = 1,476)

Risk factor^a^	Cases (*n*)	Controls (*n*)	OR^b^	95 % CI
Beer^c^				
Complete abstainers	107	143	1.00	Referent
Beer lifetime abstainers	169	324	0.70	0.48, 1.02
>0–≤0.97 g/day	105	166	0.94	0.63, 1.39
>0.97–≤3.64 g/day	59	165	0.52	0.33, 0.82
>3.64 g/day	74	164	0.62	0.39, 0.98
*p* trend^d^				0.021
Wine^c^				
Complete abstainers	107	143	1.00	Referent
Wine lifetime abstainers	70	124	0.67	0.42, 1.06
>0–≤0.78 g/day	144	232	0.79	0.53, 1.16
>0.78–≤3.29 g/day	105	231	0.69	0.46, 1.03
>3.29 g/day	88	232	0.61	0.40, 0.93
*p* trend^d^				0.031
Liquor^c^				
Complete abstainers	107	143	1.00	Referent
Liquor lifetime abstainers	49	120	0.70	0.43, 1.13
>0–≤0.80 g/day	137	232	0.86	0.59, 1.25
>0.80–≤2.94 g/day	109	233	0.59	0.40, 0.88
>2.94 g/day	112	234	0.66	0.43, 1.02
*p* trend^d^				0.059

^a^For those who drink alcohol, tertiles are based on the distribution of the drinking controls in a given type of drink

^b^Adjusted for age at reference, nulliparous (vs. multiparous), HRT and menopausal hormones, rural residential status (vs. urban), hypertension, weight at reference, waist circumference, smoking status, intake of other types of alcohol (beer/wine/liquor as a continuous variable)

^c^ *p* values were estimated for the likelihood ratio test of the overall importance of a categorical variable. For alcohol intake by tertiles, *p* = 0.012, *p* = 0.16, *p* = 0.069 for beer, wine, and liquor models, respectively

^d^ *p* trend represents the significance of a test for trend across complete abstainers and all tertiles of alcohol consumption that excludes the drink-specific abstainers

There was no evidence for effect modification of these risk estimates by BMI, lifetime physical activity, or HRT use/menopausal status (data not shown). There was also no difference in the association between alcohol intake and each specific type of endometrial cancer (data not shown).

We also examined risk estimates by the age period in which alcohol was consumed (≤17, 18–34, 35–50, ≥51 years of age). In multivariable analysis, treating alcohol intake as a continuous variable (1 g/day increments), alcohol intake was not significantly associated with endometrial cancer risk among drinkers for any of the age periods examined. Similarly, in 6.8 g/day increments (~1/2 drink/day), alcohol analyzed as a continuous variable was not statistically significant in multivariable models, with adjusted ORs (95 % CI) estimated as: 0.93 (0.74, 1.16), 0.96 (0.87, 1.07), 0.98 (0.89, 1.09), and 0.96 (0.86, 1.07) for the age periods ≤17, 18–34, 35–50, and 51+ years. When alcohol consumption was analyzed by tertiles within each age period, decreased risks were observed across all age periods; ORs were statistically significant for only the middle (≤17, 18–34, 35–50 years) or upper (18–34, 51+ years) tertiles of consumption.

## Discussion

This large, population-based case–control study conducted in Alberta, Canada, observed a statistically significant 30 % decreased risk of endometrial cancer for current drinkers compared to lifetime abstainers, and a statistically significant 29 % decreased risk for ever drinkers versus lifetime abstainers. Our data showed that alcohol consumption averaged over the lifetime in the range of 3–8 g per day (approximately 1/4 to 1/2 drinks per day in Canada) was associated with a reduced risk of endometrial cancer. A trend of decreasing risk of endometrial cancer with increasing average lifetime intakes of alcohol was similarly observed for beer, liquor, and wine consumption. No evidence was found for effect modification by the other factors examined.

To date, three meta-analyses of alcohol intake and endometrial cancer risk have been described in the literature [30, 48, 49] generally showing no association or an increased risk with relatively high levels of alcohol consumption. The vast majority of studies included in these meta-analyses did not examine lifetime alcohol intake. In the largest meta-analysis that included data from 20 case–control and seven cohort studies, Turati et al. [30] found no evidence of an association when comparing drinkers to nondrinkers (RR = 0.95, 95 % CI = 0.88–1.03) and only a slightly increased risk of endometrial cancer for heavy drinkers (≥14 drinks/week; ≥2 drinks/day) compared to nondrinkers that was not statistically significant (RR = 1.12, 95 % CI = 0.87–1.45). There was some suggestion of a weak positive association among very high alcohol drinkers particularly after menopause. In our own study, the fact that very few women were heavy drinkers within each menopausal group prohibits us from reliably corroborating those results. In a second meta-analysis of seven prospective cohort studies conducted by Friberg et al. [48], it was found that women who consumed >2.5 drinks/day compared with nondrinkers increased their risk of endometrial cancer by 25 % (95 % CI = 0.98–1.58), but again this increase was not statistically significant. Lastly, Sun et al. [49] found no evidence for the association when comparing ever-to-never alcohol consumption in six cohort (RR = 1.04, 95 % CI = 0.91–1.18) and 14 case–control studies (OR = 0.89, 95 % CI = 0.76–1.05), but did show a statistically significant increased risk with liquor consumption (RR = 1.22, 95 % CI = 1.03–1.45) based on an analysis of seven studies that reported on the type of alcohol. Our study did not support the latter analysis, showing instead inverse associations with liquor consumption averaged over the lifetime.

A key consideration when comparing our findings to others in the literature may be the level of alcohol consumed. Our study population reported a relatively low level of alcohol consumption compared to many previous studies. In a dose–response meta-analysis by Turati et al. [30], the risk of endometrial cancer was essentially unaltered by alcohol intake until consumption reached approximately 30 g/day (slightly more than 2 drinks/day), at which point the risk increased (pooled RR vs. nondrinkers = 1.39, 95 % CI, 0.89–2.19 for 38 g/day or approximately 3 drinks/day). In another dose–response meta-analysis by Friberg et al. [48], however, it was shown that small doses of alcohol, up to 1 drink/day (the range examined in our study), may be weakly and nonsignificantly protective, whereas higher doses of more than 2 drinks/day (>26 g/day of alcohol) increased endometrial cancer risk nonsignificantly. The authors described this dose–response relation, between 0 and >2.5 drinks/day, as a J-shaped curve, albeit a shallow one. In our own data, we found no evidence that mean daily alcohol intake behaved nonlinearly (*p* value for nonlinear terms = 0.245), although the overall range of alcohol consumption was relatively narrow. Further insight into the effect of alcohol dose can be gained by comparison with individual study results. When we limit a comparison of our results only to previously reported risk estimates that were based on similar levels of alcohol intake (comparing risk estimates across categories of alcohol consumption that included 0 to ~13 g/day alcohol intake or ≤1 drink/day), either there was no statistically significant association [7, 8, 10, 17, 23–27, 29–33, 35, 36, 50] or there was a statistically significant protective effect from alcohol, in both cohort [9, 15] and case–control studies [11, 12, 14, 16]. Therefore, overall, earlier studies generally suggest that low levels of alcohol intake (<1 drink/day) may have no effect on endometrial cancer risk, or low intake may have a protective effect, consistent with our results.

There are likely other reasons for inconsistent findings across studies, including the method of exposure assessment. While some studies have used very crude measures of alcohol intake based on consumption patterns at a single point in time, others have estimated alcohol consumption patterns more comprehensively. To our knowledge, only four previous studies have attempted to assess lifetime alcohol consumption and endometrial cancer risk [25, 26, 29, 36]. Most recently, lifetime alcohol intake and endometrial cancer risk were described in the context of the EPIC study, a large prospective study of over 300,000 women who were followed on average for 11 years [36]. No association was found between endometrial cancer risk and average lifetime consumption of alcohol, or with alcohol intake at age 20; former drinkers versus average lifetime intake of 0.1–6 g/day was associated with an increased risk that was not statistically significant (HR = 1.28, 95 % CI 0.98–1.68). No suggestion of dose–response trends was observed across lifetime alcohol categories ranging from 0 to >36 g/day, and as in our study, no evidence of effect modification was found. In a case–control study, Swanson et al. [29] found no evidence of an association between alcohol consumption during adulthood and endometrial cancer risk, although a statistically nonsignificant protective effect of >4 drinks/week or approximately ½ drink/day (adjusted RR vs. no alcohol = 0.34, 95 % CI 0.10–1.16) was observed for women under 55 years of age. Newcomb et al. [26] examined endometrial cancer risk in relation to participants’ alcohol intake 5 years before the interview and also in their 20 s but found no statistically significant association in either time period; however, a significant inverse association was observed in a small group of pre-menopausal women who, 5 years before the interview, consumed 1 drink/day or more. In an article by McCann et al. [25], it was unclear which life period was represented for estimated alcohol intake.

In our own study, we did not demonstrate that the time of life in which alcohol was consumed was important with respect to endometrial cancer risk. Through our age period-specific multivariable models, we observed similar risk estimates across four age periods since most women in our study, who generally consumed low amounts of alcohol, likely maintained a fairly consistent alcohol intake over their lifetimes (within most individuals, we observed only minor increases in consumption over these four age periods; data not shown). Our finding that ‘ever,’ ‘current,’ and ‘former’ (potentially early life) drinkers in our study experienced lower risks of endometrial cancer relative to lifetime abstainers supports a hypothesis that light alcohol consumption at any time in life may be beneficial. Our age period-specific analyses provide valuable insight that is lacking in our analysis of average lifetime alcohol intake, a variable that does not account for changing consumption patterns over time. However, it is also important to note that none of our age-specific analyses controlled for the effect of changing exposure over time.

Further considerations when comparing our findings to previous studies include adjustment for confounders and the source of study cases and controls. Important confounders to consider in the relation of alcohol and endometrial cancer include oral contraceptive use, menopausal status, physical activity, smoking, and fruit or vegetable intake. Failure to adjust for these may have lead to inaccurate effect estimates in some studies. Furthermore, among case–control studies, study designs have included both hospital-based and population-based studies like ours. The population-based design minimizes the possibility for selection bias and may therefore produce more accurate results. When stratifying case–control studies in their meta-analysis, Turati et al. [30] found that data from population-based studies showed a statistically significant decreased risk of endometrial cancer for drinkers compared to low/nondrinkers, while data from hospital-based studies was inconclusive.

It is biologically plausible that alcohol consumption is associated with endometrial cancer risk. While epidemiologic studies have demonstrated unfavorable changes in sex hormone levels with alcohol intake [2–6], and estrogens may be related to increased endometrial cancer risk through inducing mitotic activity, DNA replications, and mutations [51], this mechanism does not support our findings of reduced endometrial cancer risk among lifetime drinkers. Another possible mechanism that would support our findings involves the insulin response: alcohol consumption has been associated with improved insulin sensitivity and decreased fasting insulin levels [52], both of which have been associated with endometrial cancer risk, including in our study population [53]. Furthermore, alcohol may increase adiponectin levels [54] that are inversely related to insulin resistance. To further support this hypothesis, the growth of endometrial cells in vitro is stimulated through insulin, which binds to insulin receptors in the endometrium [55]. These opposing mechanisms, whereby alcohol may act as an effect modifier in the hypothesized causal association between insulin exposure and endometrial cancer risk, may help to explain some contradictory findings in the literature to date.

Certain limitations of our study and statistical analyses must be considered when interpreting our findings. The retrospective case–control design of our study can be prone to measurement error due to the difficulty in recalling past behavior, particularly over the lifetime. To minimize this error in our study, all interviewers were trained in cognitive interviewing methods and used lifetime calendars as recall aids to help respondents recall their lifetime alcohol consumption. Social desirability bias is also possible in our data given that alcohol intake was the exposure of interest, i.e., some women may have underreported their intake. In addition, a healthy volunteer bias may have occurred given the lower response rate among the controls and their slightly higher educational levels and overall health. Although, in comparison with a general population sample of Canadians that we made for this study population [37], we concluded that no major selection bias was evident for our control population. Finally, our capacity for subgroup analyses of our data was limited by relatively small numbers of cases and controls in some categories of women; therefore, we may have not had sufficient power to detect some statistically significant associations.

In conclusion, this population-based study provides evidence for a protective association between alcohol consumption over the lifetime and endometrial cancer risk. Our data were suggestive that <1 drink of alcohol per day averaged over the lifetime is associated with a reduced risk of endometrial cancer, which is consistent with a number of previous studies examining similar levels of alcohol intake. These findings do not warrant advising increased alcohol consumption to reduce the risk of endometrial cancer as alcohol consumption may increase risk of several other important diseases including other cancers.
